# Deep Learning-Based Channel Estimation Techniques Using IEEE 802.11p Protocol, Limitations of IEEE 802.11p and Future Directions of IEEE 802.11bd: A Review

**DOI:** 10.3390/s26051658

**Published:** 2026-03-05

**Authors:** Saveeta Bai, Jeff Kilby, Krishnamachar Prasad

**Affiliations:** School of Engineering, Computer and Mathematical Sciences, Auckland University of Technology, Auckland 1010, New Zealand; saveeta.bai@autuni.ac.nz (S.B.); krishnamachar.prasad@aut.ac.nz (K.P.)

**Keywords:** channel estimation, IEEE802.11p, V2V, deep learning 802.11bd VANET, DSRC, IEEE802.11bd

## Abstract

Vehicular communication networks demand highly efficient and accurate channel estimation to ensure reliable data exchange in high mobility scenarios. The IEEE 802.11p standard is widely regarded as the foundation of the Vehicle-to-Vehicle (V2V) communication channel; however, it is constrained by limited pilot resources and a fixed pilot structure, which degrade the performance and effectiveness of traditional estimation techniques, particularly in dynamic environments. Recent advances in deep learning offer significant potential for addressing these issues by improving estimation accuracy and modelling complex channel dynamics. Though deep learning-based methods introduce trade-offs in computational complexity and accuracy, these are crucial constraints in latency-sensitive V2V scenarios. This article presents a comprehensive review of deep learning-based channel estimation techniques, analysing methods for the IEEE 802.11p standard and critically examining their limitations in both classical and deep learning-based approaches. Additionally, the article highlights improvements introduced by IEEE 802.11bd, which features an enhanced pilot structure and advanced modulation schemes, providing a more robust framework for adaptive, efficient channel estimation. By identifying future research pathways that balance delay, complexity, and accuracy, an intelligent and effective transportation system can be established.

## 1. Introduction

According to the World Health Organisation (WHO), traffic accidents cause around 1.2 million deaths worldwide annually, or almost one-fourth of all injury-related deaths [1,2]. In addition to the terrible death toll, traffic-related incidents cause injuries to nearly 50 million people each year. These worrying outcomes emphasise the pressing need for a transformation in transport management and traffic safety. Deploying Intelligent Transportation Systems (ITS) is one of the most promising solutions to address this rapidly growing problem. At the core of ITS is Cooperative Vehicular Communication Systems (CVCS) [3], which facilitate real-time data exchange through V2V [4,5,6,7], Road-to-vehicle (R2V), vehicle-to-infrastructure (V2I), and vehicle-to-network (V2N) communication, enabling proactive responses to potential hazards. Vehicle-to-Everything (V2X) communication plays a key role by integrating these interactions into a unified framework, thereby enhancing safety, traffic efficiency, and connectivity [4,5]. While safety remains the main driver, ITS also supports dynamic route optimisation, real-time congestion management, vehicle performance analysis, and infrastructure evaluation. The evolution of Cooperative ITS (C-ITS) highlights a global push toward connected mobility, aiming to reduce accidents, ease congestion, improve fuel efficiency, and support sustainable urban planning [6]. Overall, ITS plays a key role in transforming transportation into a safer, more innovative environment [7].

Since there is a rising need to improve road safety, traffic efficiency, and overall transportation management, Vehicular Ad Hoc Networks (VANETs) are considered a key ingredient in allowing the advancement of ITS [8]. VANETs are a specific form of mobile ad hoc network (MANET) designed to support dynamic wireless communication among vehicles (V2V), between vehicles and infrastructure (V2I), and across broader network systems (V2X). They enable the real-time exchange of information, such as traffic conditions, hazard alerts, and navigation updates, thereby allowing vehicles to make timely and informed decisions. This self-organising, decentralised communication approach improves situational awareness, reduces collision risk, and supports traffic control.

Furthermore, VANETs are needed to create safe, creative, and effective ITS infrastructures because they are the foundation for integrating cutting-edge technologies like edge computing, software-defined networking (SDN), and the Internet of Vehicles (IoV) [9]. VANETs are broadly classified based on the communication technologies they use. One category depends on cellular networks, including 4G, 5G, and upcoming 6G, offering huge coverage and high data rates [10,11]. Another category is based on IEEE 802.11 protocols (e.g., 802.11p, 802.11bd), which are specifically designed for low-latency, short-range vehicular communication [12]. Both approaches play a significant role in ensuring reliable and efficient ITS operations. The proposed research focuses on IEEE 802.11p and IEEE 802.11bd protocols, which are specifically designed for vehicular communication under the Dedicated Short-Range Communication (DSRC) standard [13]. These protocols offer low latency, direct V2V and V2I communication, and independence from cellular infrastructure, making them highly suitable for safety-critical applications in ITS. IEEE 802.11p has been widely adopted in early ITS deployments, while IEEE 802.11bd, as its enhanced successor, supports higher data rates, backward compatibility, and robust performance in high-mobility scenarios. Focusing on these protocols allows the research to target reliable, low-latency communication tailored for real-time vehicular environments.

However, maintaining reliable communication is severely hampered by the highly dynamic nature of vehicle environments, which are characterised by high mobility, rapidly shifting topologies, and multipath fading. Because it enables the receiver to precisely simulate and adapt to wireless channel conditions, channel estimation is essential. Achieving robust performance in high-speed vehicular scenarios, especially under IEEE 802.11p and its advanced successor, IEEE 802.11bd, requires effective channel estimation because it improves channel performance, reduces Mean Squared Error (MSE) and Bit Error Rate (BER), and increases overall link reliability. Established channel estimation techniques have played a fundamental role in ensuring reliable wireless communication, particularly in vehicular environments where the channel is highly dynamic due to mobility-induced Doppler effects and multipath fading. Among these, the Least Squares (LS) estimator is the most basic and widely used due to its simplicity and low computational complexity; however, it is not robust under low SNR and fast-fading conditions. To address these limitations, more advanced techniques have been developed, including data-pilot-aided (DPA), Spectral Temporal Averaging (STA), and Constructed Data Pilots (CDP), which aim to enhance performance by leveraging past decisions or averaging strategies. Enhanced versions, such as Iterative CDP (iCDP) and Time-Domain Reliable Test Frequency-Domain Interpolation (TRFI), offer greater adaptability to channel variations. Techniques like Enhanced TRFI (E-TRFI) and MMSE-based approaches (e.g., MMSE-VP, LMMSE) further incorporate statistical channel knowledge to minimise estimation error. Recent hybrids, such as Adaptive STA-MMSE-VP, aim to balance performance and complexity, underscoring the ongoing evolution of channel estimation methods to meet the demands of high-mobility vehicular communication systems. Conventional channel estimation methods are widely used but have many drawbacks, particularly in high-mobility vehicle environments. The frequently evolving and intricate structure of vehicle wireless channels is not adequately captured by these techniques, which often rely on oversimplified statistical models and assumptions like channel stationarity and linearity. As a result, their performance degrades in scenarios involving severe Doppler shifts, sparse pilot configurations, and non-line-of-sight conditions. Furthermore, advanced estimators such as LMMSE, while more accurate, require substantial computational resources and prior knowledge of channel statistics, making implementation challenging in highly dynamic environments.

To address these issues, deep learning (DL)-based channel estimation has emerged as an effective alternative [14,15]. Without relying on predetermined assumptions, deep learning models, such as convolutional neural networks (CNNs) and long short-term memory (LSTM) networks, can directly learn intricate channel patterns from input. These models are ideal for dynamic vehicular settings because they are robust, exhibit minimal latency and generalise better to unknown channel conditions. More recently, hybrid deep learning techniques, such as CNN + LSTM or Transformer + ResNet, have demonstrated better performance than standalone models by combining the strengths of multiple architectures. These techniques capture both spatial and temporal relationships in the channel. Since latency is a crucial limitation in vehicular communication, achieving a workable solution requires careful trade-offs among estimation efficiency, processing complexity, and latency. Hence, there is a desperate need to develop adaptive, lightweight hybrid models that can balance these three factors, ensuring reduced computational complexity and low latency without compromising channel accuracy.

The channel estimation algorithms in this context have been examined in numerous previous studies and literature reviews. These studies frequently assess both traditional and deep learning-based approaches, emphasising gains in precision, resilience, and flexibility in vehicle environments. In addition, future directions, including cutting-edge technologies such as 5G and 6G for vehicular communications, have also been mentioned in several evaluations. A comprehensive, targeted assessment of channel estimation strategies for IEEE 802.11p-based VANETs remains conspicuously lacking. Despite its widespread use in V2V communication, IEEE 802.11p has significant drawbacks, including limited support for high-mobility scenarios, sensitivity to Doppler effects, and sparse pilot configurations. In addition, existing studies lack a balanced approach to performance metrics, including accuracy, computational complexity, and delay. Thus, the objective of this research is to provide a comprehensive and systematic review of Deep learning-based channel estimation techniques tailored for IEEE 802.11p protocols, critically analysing their strengths and shortcomings. Furthermore, this work examines how these limitations are being addressed as IEEE 802.11bd evolves. It assesses the alignment of IEEE 802.11bd with the proposed Transformer Neural Networks, offering a forward-looking perspective on future V2V communication systems.

The remainder of the article is structured as follows. Section 2: background information on channel estimation, traditional and deep learning channel estimation techniques, background on VANET, and details of the IEEE 802.11p and IEEE 802.11bd protocols, including their frame structures, have been discussed. Section 3 provides a systematic review of deep learning-based channel estimation schemes for the IEEE 802.11p protocol. A future vision for using IEEE 802.11bd for channel estimation techniques is presented in Section 4. Finally, the paper concludes with a discussion and perspectives on future directions.

## 2. Background

**Figure 1:**
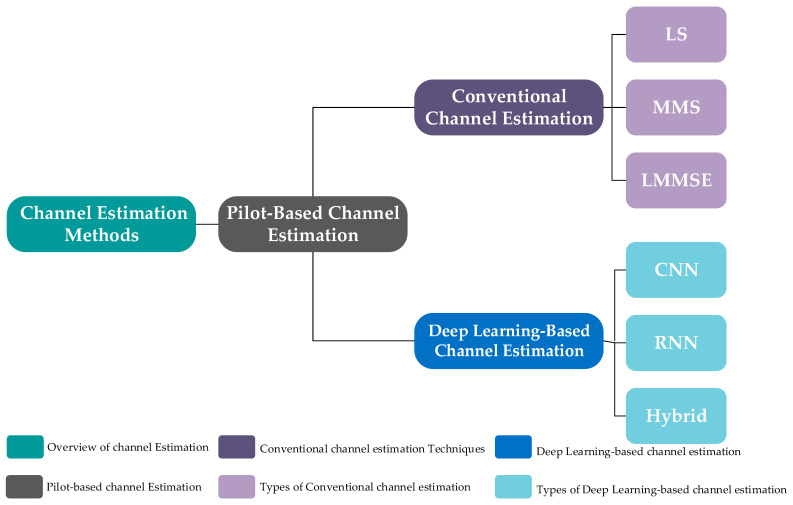
Classification of Channel Estimation Methods.

This background presents fundamental concepts of channel estimation, including the system model and conventional techniques, and subsequently discusses deep learning-based channel estimation techniques in detail. The classification of channel, traditional channel estimation, and deep-based channel estimation is shown in Figure 1 and will be discussed in detail. This offers a structured comparison of these approaches. Finally, the background of VANETs and the system descriptions of IEEE 802.11p and IEEE 802.11bd, including their advancements, types, and protocols, will be discussed in detail.

### 2.1. Background of Channel Estimation

Determining the characteristics and features of a communication channel is known as channel estimation. It helps alleviate channel-induced distortions, thereby improving the reliability and performance of wireless communication systems.

In addition, channel estimation is a crucial technique for OFDM modulation in wireless communication systems. Orthogonal frequency division multiplexing (OFDM) is used in many wireless communication systems due to its exceptional bandwidth efficiency, high transmission rate, and long-term reliability in the presence of multi-path fading and delay [16,17]. To transform a frequency-selective channel into a non-frequency-selective channel, OFDM is used to create orthogonal narrowband subchannels from the current spectrum’s multiple overlapping bands [18]. Additionally, the technique of cyclic prefix (CP), which is accomplished by extending an OFDM symbol that contains a portion of its head or tail, results in negligible inter-symbol interference (ISI) in OFDM [4]. OFDM is widely utilised in wireless communication because of these advantages.

#### 2.1.1. System Model of Channel Estimation

**Figure 2:**
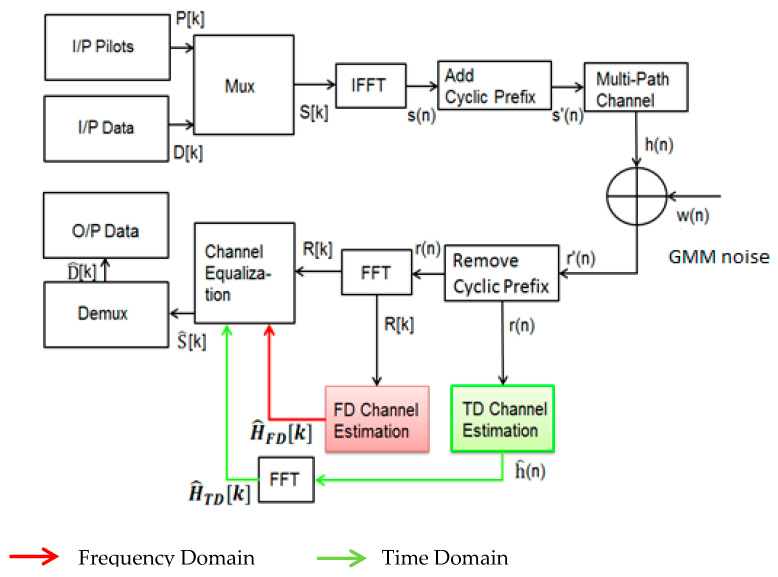
System block diagram of Chan nel Estimation [19].

The System Model for channel estimation in the frequency domain (FD) and time domain (TD) is shown in Figure 2 [19].

The model starts with the generation of pilots and data subcarriers $P\left[K\right]$ and $D\left[K\right]$, correspondingly. Next, these subcarriers are added to the mux block and give the frequency-domain samples $X\left[K\right]$. Then, the Inverse Fast Fourier Transform is applied on $X\left[K\right]\;$ samples that transform FD samples $X\left[K\right]$ into TD $x\left(n\right)$, which is given in (1).

(1) $$x\left(n\right)=IFFT\left\{X\left[K\right]\right\}=\sum_{k=0}^{N-1}X\left[K\right]e^{\frac{j2\pi kn}{N_{FFT}}}$$

where $N_{FFT}$ refers to the number of Fast Fourier Transforms. To remove inter-symbol interference, a cyclic prefix $N_{g}$ are inserted in each OFDM symbol, and samples become $x’\left(n\right)$, that can be stated as (2).

(2) $$x’\left(n\right)=\left\{\begin{array}{c} x\left(N_{FFT}+n\right),\;\;\;n=-N_{G}+1\ldots ,-1\\ x\left(n\right),\;\;\;\;\;\;\;\;\;\;n=0,1,\ldots \ldots ,N_{FFT}-1 \end{array}\right.$$

After passing through the frequency-selective multipath channel, the received signal becomes (3).

(3) $$y’\left(n\right)=x’(n)⨂h\left(n\right)+w(n)$$

The received signal is y’(n) with the cyclic prefix that would be $y\left(n\right)$ after removing the cyclic prefix. When FFT  is applied to Equation (3), it is rewritten as (4):

(4) $$Y[k]=FFT\left\{y\left(n\right)\right\}=\frac{1}{N_{FFT}}\sum_{n=0}^{N_{FFT}-1}y(n)e^{\frac{j2\pi kn}{N_{FFT}}}$$

$$n=1,2,\ldots .N_{FFT}-1$$

Suppose there is no ISI because the length of CIR is smaller than the guard band interval. The response of $Y\left[k\right]$ can be represented in (5):

(5) $$Y[k]=X\left[k\right]H[k]+W[k]\;0\leq k\leq N_{FFT}-1$$

where *X* and *Y* denote the transmitted and received FD signals at each subcarrier, respectively.

#### 2.1.2. Conventional Channel Estimation Techniques

Conventional channel estimation techniques rely on well-defined mathematical models and pilot-based approaches to characterise the channel response. These traditional techniques have been widely applied to wireless communications systems due to their simple mathematical modelling and analytically predictable performance.

##### Least Square Estimator

The Least Square (*LS*) channel estimation technique is a simple, low-complexity method that estimates the channel using known pilot signals. The criterion of the *LS* method is to minimise the weighted sum of squared errors between the estimated and actual values.

Consider a system model in the FD for OFDM systems as shown in Equation (5).

However, *H* and *W* in Equation (5) represent the channel transfer function and AWGN noise, respectively, for the OFDM signal. The transfer function ${\hat{\;H}}_{LS}\left[k\right]$ The *LS* channel estimation is calculated as (6) [20,21].

(6) $${\hat{H}}_{P,LS}\left[k\right]={\left[\frac{Y_{P}\left(N_{F}c\right)}{X_{P}\left(N_{F}c\right)}\right]}^{T}\;\;\;\;\;\;\;\;\;\;\;\;\;\;\;\;0\leq c\leq N_{P}-1$$

where  T represents the transposition of the matrix; $X_{P},Y_{P}$ are the inputs and output values of the pilot subcarrier, respectively, $N_{F}$ is the pilot spacing and $N_{P}$ is the total number of pilots.

##### Minimum Mean Square Error (MMSE) Estimator

The *MMSE* estimator is more accurate than the LS estimator, which incorporates noise statistics and channel correlation; however, the *MMSE* estimator requires higher computational resources.

The difference between the estimator and the estimated value may be expressed as in (7), the average of the squares, which is the MSE:

(7) $$e=H-\hat{H}$$

The mean squared error (MSE) between the estimator and the estimated value is as follows in (8):

(8) $$E\left\{{\left|e\right|}^{2}\right\}=E\left\{{\left|H-\hat{H}\right|}^{2}\right\}$$

One way to simplify the *MMSE*-based channel estimation is as in (9), [19]:

(9) $${\hat{H}}_{MMSE}=R_{HH}(R_{HH}+\sigma_{N}^{2}{{\left(X^{H}X\right)}^{-1})}^{-1}{\hat{H}}_{LS}$$

where the auto-covariance matrices of H is denoted by $R_{HH}$. The performance of the *MMSE* estimator is superior to that of the *LS* estimator.

##### Linear Minimum Mean Square Error Estimator

Linear Minimum Mean Square Error (LMMSE) is an advanced statistical method used to estimate the channel response by minimising the MSE between the estimated and actual channel responses. Compared with the *LS* algorithm, which is entirely dependent on a known pilot symbol, the LMMSE algorithm integrates prior knowledge of both channel statistics and noise variance. LMMSE leverages the channel’s autocorrelation to obtain more precise and robust estimates, particularly at low *SNR*. However, because of the matrix inversion, this improvement significantly increases computational complexity, making it less suitable for systems with strict latency constraints.

The mathematical expression of LMMSE is given in Equation (10).

(10) $${\hat{H}}_{MMSE}=R_{HH}(R_{HH}+\frac{\beta }{SNR}I{)}^{-1}\hat{H_{LS}}$$

#### 2.1.3. Deep Learning-Based Channel Estimation Techniques

Deep learning has emerged as a critical component of modern wireless communications, owing to its ability to learn complex channel dynamics and adapt to rapidly changing conditions. Compared with conventional estimators that rely on rigorous mathematical assumptions, deep learning models can directly extract channel characteristics from data, providing more precise and robust estimates. Conventional channel estimation methods, such as *LS* and *MMSE*, rely heavily on accurate channel statistics and degrade significantly when pilots are limited, the cyclic prefix is removed, or nonlinear distortions occur. Conversely, deep learning approaches address these problems by learning channel characteristics end-to-end, thereby enhancing robustness and performance even under adverse conditions. The deep learning channel estimation techniques are discussed in detail in the following subsections.

##### Convolutional Neural Networks-Based Channel Estimation

**Figure 3:**
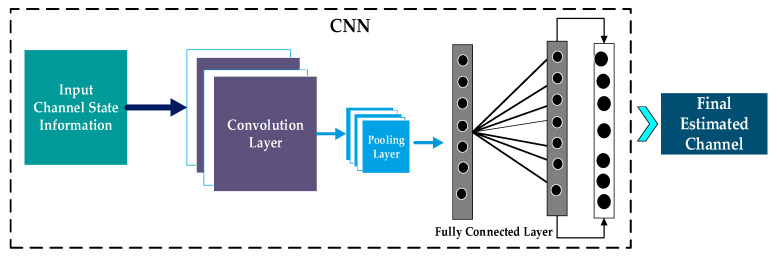
CNN-Based C.E. System Architecture.

Due to their strong ability to capture spatial correlations, Convolutional Neural Networks (CNNs) have become the most widely used for channel estimation, as shown in Figure 3. By applying feature-extraction filters, CNN-based estimators capture key channel characteristics, such as sparsity, smoothness, and delay-Doppler structure, without relying on precise channel models. The CNN architecture for channel estimation consists of the following layers: input, Convolutional, Pooling, fully connected, and the final estimated channel.

- **Input: Channel State Information** 

The CNN receives raw Channel State Information (CSI) as a two-dimensional matrix across the time and frequency domains. This representation captures spatial and temporal correlations in the channel.

- **Convolutional Layer** 

A CNN’s convolutional layer is its fundamental component. The convolutional layer uses adaptive kernels on the CSI to extract key channel features, including multipath structure, delay-Doppler fluctuations, and inter-subcarrier correlations. This layer converts the raw CSI into a more informative representation compatible with channel modelling by sliding across the input and generating feature maps.

- **Pooling Layer** 

To reduce dimensionality and computational complexity, the pooling layer down-samples the feature maps. In channel estimation, pooling preserves dominant responses while suppressing noise and redundant information, thereby strengthening the extracted channel features.

- **Output: Final Estimated Channel** 

After passing through all the layers, the system outputs the final estimated channel, which is more accurate and robust than conventional channel estimation techniques.

##### Recurrent Neural Networks-Based Channel Estimation

**Figure 4:**
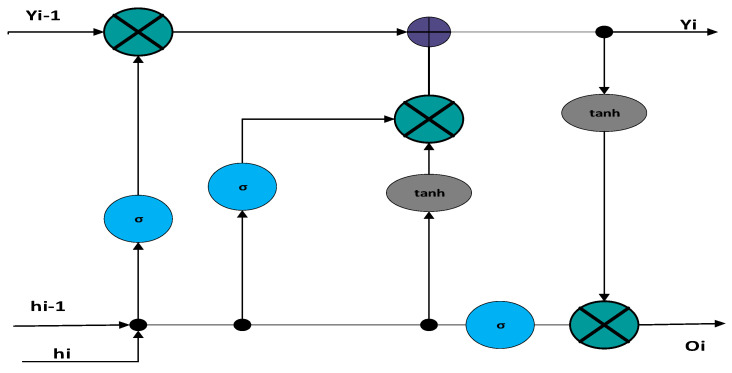
LSTM-Based C.E. System Architecture.

The LSTM (Long Short-Term Memory) technique within Recurrent Neural Networks (RNN) utilises feedback connections to operate like a general-purpose computer. This method is applied in sequence analysis, pattern recognition, and image processing. An LSTM comprises three primary components: the input gate, output gate, and forget gate. These elements enable the LSTM to remember computations from previous time steps and regulate the influx of input into the neuron, as depicted in Figure 4. One of the key advantages of the LSTM approach is its ability to determine these processes solely from the current input. The LSTM network topology shown in the figure comprises three gates: the input, forget, and output gates. Additional memory cells, in the same form as the hidden state, store further data. The previous cell’s data is saved or discarded depending on the forgotten gate. The input gate handles any extra data that must be introduced to the cell state. Finally, the output must be recognized. This is a filtered version based on the current cell state.

##### Hybrid Channel Estimation

**Figure 5:**
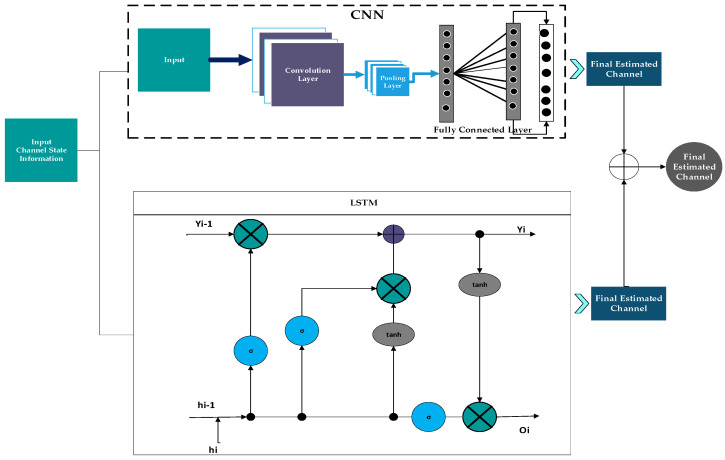
Hybrid Channel Estimation.

A new hybrid technique is proposed for efficient channel estimation, with improved performance metrics, including MSE and SNR. Several deep learning techniques have been proposed to improve channel estimation, but they are computationally intensive. Thus, CNN is integrated with an LSTM to address computational complexity and time-consuming issues. CNNs are easy to develop and require less computational time. Moreover, the hidden neurons in the convolutional layer are effectively realised using this CNN. However, CNN requires additional training for channel estimation. Hence, there is a risk of system overburden and a probability of error. LSTM classifiers are widely used for channel estimation owing to their straightforward architecture. But the LSTM system is highly complex. To avoid unnecessary training, computational, and time complexity, the hybrid model is developed by integrating CNN and LSTM architectures. Here, the signal is taken as input. The layers of a CNN, such as pooling, convolutional, and fully connected layers, are defined. This is used to extract the channel at the acceptor side when the channel is estimated from the LS technique. This demodulation process is performed using a developed hybrid system architecture, and the proposed algorithm improves its efficiency. Finally, the hybrid system architecture improves overall performance by reducing BER, MSE and SNR. Figure 5 shows hybrid channel estimation techniques [22].

### 2.2. Background of Vehicular Ad Hoc Networks (VANET)

VANETs are a subset of Mobile Ad Hoc Networks (MANETs) designed to enable wireless communication among vehicles (V2V and R2V) and, more broadly, V2X communication. The V2X paradigm encompasses V2V, V2I, V2P, and V2N interactions, providing a comprehensive framework for real-time data exchange. VANETs serve as the communication backbone of ITS, aiming to improve road safety, traffic efficiency, and the overall driving experience [23,24].

Early advancements in vehicle communication are where the idea for VANETs originated. The 1925 patent, “Radio Warning Systems for Use on Vehicles,” marked a significant turning point in history, as it envisioned peer-to-peer radio alerts between cars, laying the early conceptual groundwork for modern V2V communication. But thanks to developments in wireless technology, mobile computing, and the growing demand for intelligent mobility, VANETs officially became a research area in the early 2000s. During this time, the term “VANETs” referred to dynamic, self-organising vehicle networks. VANETs have been integrated into the broader V2X ecosystem as the field has evolved, and the ecosystem now offers sophisticated cooperative and autonomous driving capabilities. A crucial moment in this evolution was the Federal Communications Commission (FCC) ‘s 1999 allocation of 75 MHz in the 5.9 GHz band for ITS applications, which accelerated the development of DSRC and subsequent V2X standards.

A number of cutting-edge wireless standards have been developed to satisfy the exacting needs of ultra-reliable, low-latency communication. One of these, IEEE 802.11bd, was created especially for V2X communication and is an enhanced version of IEEE 802.11p. Because of its increased throughput, improved reliability, and backward compatibility with IEEE 802.11p, it is suitable for high-bandwidth, safety-critical vehicle applications. Meanwhile, new technologies such as 5G NR-V2X, Cellular V2X (C-V2X), and Multi-access Edge Computing (MEC) are turning VANETs into robust platforms that can enable real-time traffic coordination, autonomous driving, and cooperative sensing.

**Figure 6:**
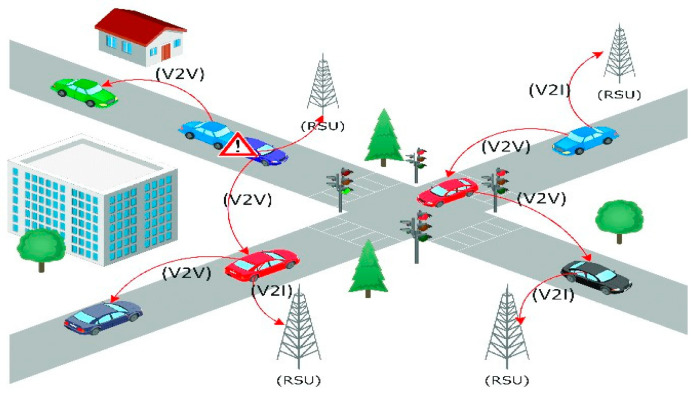
Different Types of Communication in VANET [24].

The operational framework of VANETs is based on two core hardware components: the On-Board Unit (OBU) and the Roadside Unit (RSU), as shown in Figure 6. Each vehicle is equipped with an OBU, which enables communication with other vehicles and infrastructure. OBUs collect information from the Global Positioning System (GPS), onboard sensors, and other sources to support route optimisation, traffic updates, and safety alerts. On the other hand, RSUs are stationary communication devices located beside roads or at crossroads. They serve as a bridge between automobiles and central traffic management systems, improving communication coverage and ensuring greater reliability. RSUs are essential for communicating safety messages, weather warnings, and traffic updates to surrounding vehicles. Numerous wireless technologies [25], such as DSRC, cellular networks (4G, 5G, and soon to be 6G), Zigbee [26], Satellite communication [27], WiMAX, Wi-Fi [28], Bluetooth [29] and visible light communication (VLC) [30]. These components are used to communicate. When combined, they guarantee smooth, instantaneous information sharing.

**Figure 7:**
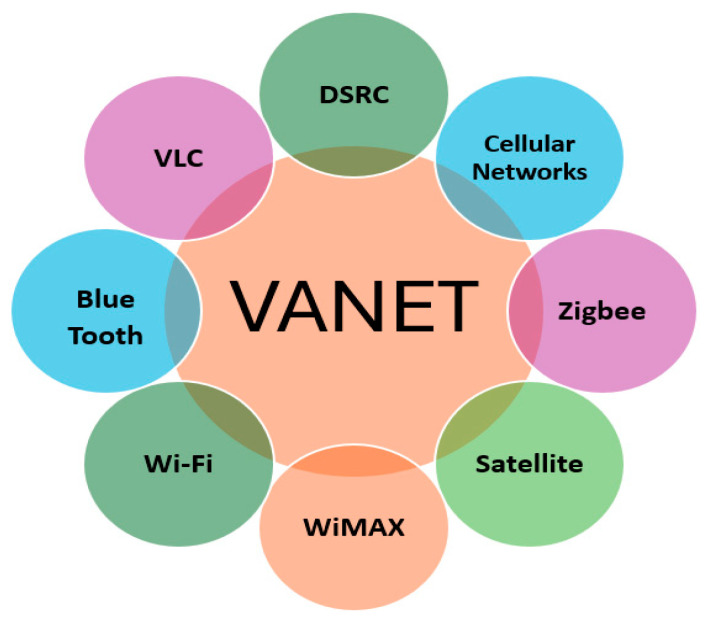
Different Wireless Technologies used in VANET communications.

VANETs rely on numerous wireless communication technologies to support different connectivity types, including V2V [31], V2I [32], and V2X. VANET systems are primarily composed of two key components: the OBU and the RSU. Each vehicle is equipped with an OBU, which enables wireless communication with other vehicles and infrastructure. OBUs collect data from vehicle sensors, GPS, and other sources to facilitate safety alerts, traffic updates, and route optimisation. On the other hand, RSUs are fixed units deployed along roads or intersections to enhance coverage and communication reliability by exchanging data between vehicles and the central traffic management system. RSUs are critical in disseminating safety messages, weather alerts, and traffic congestion updates to nearby vehicles [9,23]. These components work together using different wireless technologies, such as DSRC, Cellular Networks (4G, 5G, and 6G), Wi-Fi, and Satellite Communication, to ensure seamless information exchange, as shown in Figure 7 [8].

#### 2.2.1. Classification of VANET Technologies

**Figure 8:**
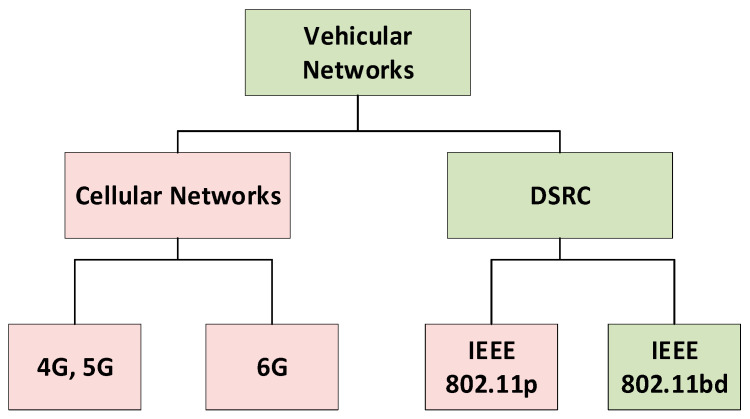
Classification of VANET.

The VANET technology classification is shown in Figure 8, with the sections highlighted in green that are pertinent to this research. Cellular networks and DSRC are the two most often used technologies for automobile communication. DSRC and Cellular Networks. 6G cellular networks, which offer improved latency, dependability, and lightning-fast connectivity for vehicle communication, have replaced 4G and 5G cellular networks. Cellular V2X (C-V2X), a notable development in this field, enables various channel models to interact, enhancing infotainment and traffic coordination. However, IEEE 802.11p and its successor IEEE 802.11bd are the main components of DSRC.

Despite its widespread use for low-latency vehicular communication, IEEE 802.11p has drawbacks, including lower data rates and less support for high-mobility situations. IEEE 802.11bd, by contrast, overcomes these limitations by enabling higher data rates, greater reliability, and greater flexibility in dynamic vehicle environments. To ensure more reliable communication systems, this classification emphasises the transition from conventional DSRC-based communication to next-generation vehicular networking technologies.

#### 2.2.2. Comparison of IEEE 802.11 Standards for VANETs

**Figure 9:**
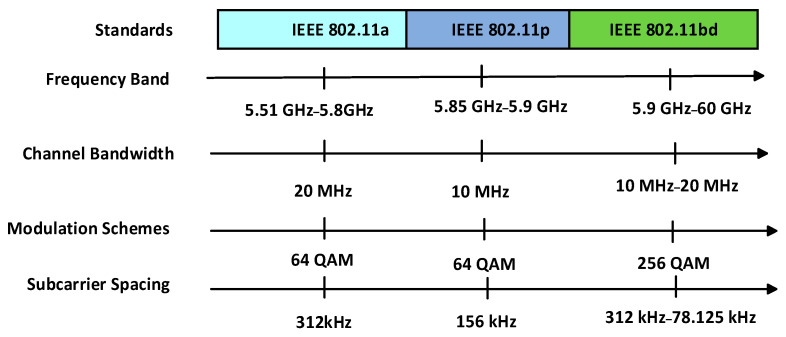
Comparison of IEEE 802.11 Standards.

The comparative analysis of PHY-layer implementations of different IEEE 802.11 standards used in vehicular networks is shown in Figure 9 [33], namely IEEE 802.11a, IEEE 802.11p, and IEEE 802.11bd, based on key parameters such as frequency band, channel bandwidth, data rate, and subcarrier spacing. IEEE 802.11a operates in the 5.51–5.8 GHz frequency band with a 20 MHz channel bandwidth, supporting data rates of 6–54 Mbps. IEEE 802.11p, specifically designed for vehicular environments, operates in the 5.85–5.9 GHz band with a 10 MHz channel width, which helps reduce interference but limits data rates to 3–27 Mbps. In contrast, IEEE 802.11bd, the latest enhancement, operates in the 5.9–60 GHz range with flexible bandwidth options of 10 MHz and 20 MHz.It significantly outperforms its predecessor by offering higher subcarrier spacing (up to 78.125 kHz), increased data rates, and improved spectral efficiency, making it a more robust and scalable solution for next-generation vehicular communication [34,35].

#### 2.2.3. System Description

This section presents the specifications, frame structure, and transmitter-receiver design of the IEEE 802.11p standard. Additionally, the evolution of IEEE 802.11p to IEEE 802.11bd is discussed. Finally, the system model for channel estimation in IEEE 802.11bd and the corresponding channel estimation schemes are described.

##### IEEE 802.11p Standard Specifications

**Figure 10:**
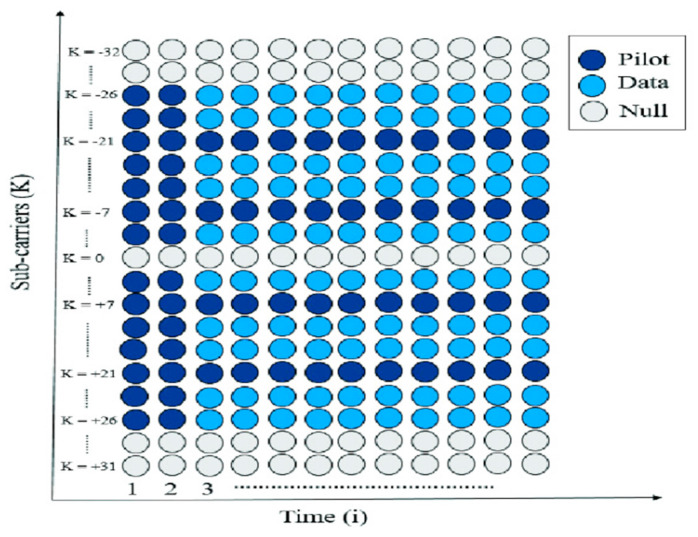
Subcarrier arrangement of IEEE 802.11p [38].

IEEE 802.11p is based on IEEE 802.11a, with enhancements required to support C-ITS applications [36,37]. In IEEE 802.11p, a 10 MHz bandwidth is used instead of the 20 MHz bandwidth in IEEE 802.11a; thus, all time-domain parameters in IEEE 802.11p are twice those in IEEE 802.11a. For instance, the doubled guard interval reduces the inter-symbol-interference (ISI) more than the guard interval in IEEE 802.11a. IEEE 802.11p also employs Orthogonal Frequency Division Multiplexing (OFDM) with 64 subcarriers, as shown in Figure 10. Only 52 are active in the range from −26 to 26, except for the 0th subcarrier, which is null. Pilots are embedded into the subcarriers for channel tracking. The other active subcarriers are used for data. The IEEE 802.11p standard enables data exchange between V2V and R2V over a range of 1 km, utilising transmission rates of 3 Mbps to 27 Mbps with various modulation and coding schemes and supports high vehicle velocities. Table 1 presents the main physical-layer parameters of IEEE 802.11p.

**Figure 11:**
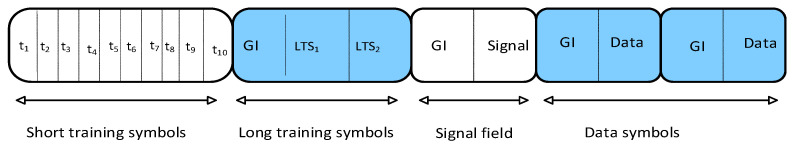
IEEE 802.11p transmitted frame structure [38].

The main physical layer parameters of the IEEE 802.11p standard are listed in Table 1. The IEEE 802.11p frame structure is shown in Figure 11, starting with the preamble field that includes (i) a short training sequence consisting of ten short sequences, each of duration 1.6 µsec, used by the receiver for signal detection and time synchronisation, and (ii) long training symbols used for channel estimation. The guard interval GI is used for the long training sequence and also used as a cyclic prefix 1 for each OFDM data symbol to mitigate ISI. Following the preamble, the signal field comprises a single CP-OFDM symbol that carries the physical layer convergence protocol (PLCP), which provides information about the payload. Finally, the data field contains a sequence of OFDM symbols corresponding to the data payload.

#### 2.2.4. IEEE 802.11p Transceiver

**Figure 12:**
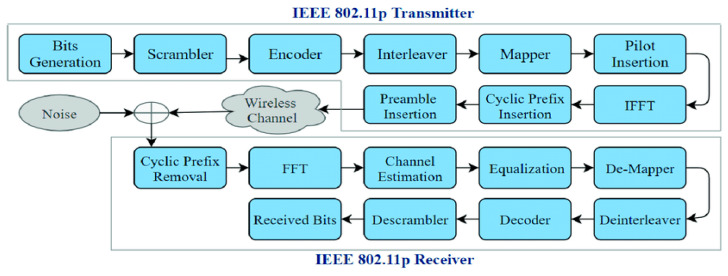
IEEE 802.11p transmitter-receiver block diagram [38].

As shown in Figure 12, the first operation on the transmitter side is the generation of binary bits. These bits are then scrambled to randomise their pattern, preventing long sequences of 1 s or 0 s. Scrambling helps the timing recovery circuit function effectively and ensures that the signal’s power spectrum is independent of the transmitted data. Next, the scrambled bits are processed by a convolutional encoder, which introduces redundancy into the bitstream. This redundancy enables error correction, allowing the receiver to mitigate channel impairments and achieve reliable communication. To combat channel noise, such as burst errors or fading, bit interleaving is applied. The interleaver rearranges the input bits to distribute consecutive bits across different blocks. This is achieved through a permutation process that maps adjacent bits to non-adjacent subcarriers, thereby improving error correction at the receiver. After interleaving, the bits are mapped according to the selected modulation scheme. The IEEE 802.11p standard supports four modulation techniques: BPSK, QPSK, 16-QAM, and 64Q-AM. Grey coding ensures that adjacent symbols differ by only one bit, reducing the probability of bit errors. Following bit mapping, the OFDM symbols are constructed. Data symbols and pilot signals are assigned to active subcarriers and passed through the IFFT block to generate time-domain OFDM symbols. A cyclic prefix is then appended to mitigate inter-symbol interference. Finally, the IEEE 802.11p packet is formed by combining the CP-OFDM symbols with predefined preamble symbols into a single frame. The preamble is used for synchronisation and initial channel estimation at the receiver. The CP is removed, and the signal is processed via FFT. Pilot subcarriers assist in channel tracking, while data symbols are sent to the equaliser. The equalised data is then demapped to retrieve the encoded bits. Subsequently, deinterleaving, decoding, and descrambling operations are performed to reconstruct the detected bits.

#### 2.2.5. IEEE 802.11bd: Advancement over IEEE 802.11p

IEEE 802.11bd is the next-generation vehicular communication standard, evolving from IEEE 802.11p to meet the needs of connected and autonomous vehicles [35]. While IEEE 802.11p has been widely adopted in VANETs due to its low latency and direct V2V communication capabilities, it faces throughput, reliability and interference limitations, particularly in dense traffic environments. IEEE 802.11bd addresses these challenges by introducing enhancements, including higher data rates, improved reliability, and backward compatibility with IEEE 802.11p. Key advancements in IEEE 802.11bd include the integration of multiple-input multiple-output (MIMO) technology, increased bandwidth, and enhanced error correction techniques. These improvements enable more reliable communication, even in high-mobility environments such as highways, ensuring better performance for safety-critical applications.

A comparative analysis of IEEE 802.11p and IEEE 802.11bd in Table 2 highlights significant differences in data rates, bandwidth, modulation schemes, and latency [39]. The table shows that IEEE 802.11bd outperforms its predecessor in spectral efficiency, packet error rate, and robustness under challenging communication conditions. IEEE 802.11bd retains the same down-clocked 10 MHz frequency band as IEEE 802.11p while introducing an optional 20 MHz operation. The outdated convolutional coding has been replaced with low-density parity-check (LDPC) channel coding, and a 256-QAM modulation option has been introduced for high-throughput applications [33]. To address doubly selective channels with strong Doppler shifts, preamble-based channel estimation has been enhanced by employing high-density midambles. Additionally, dual carrier modulation (DCM) and range extension mode, derived from IEEE 802.11ax, have been proposed as optional features to increase transmission range [40,41].

**Figure 13:**
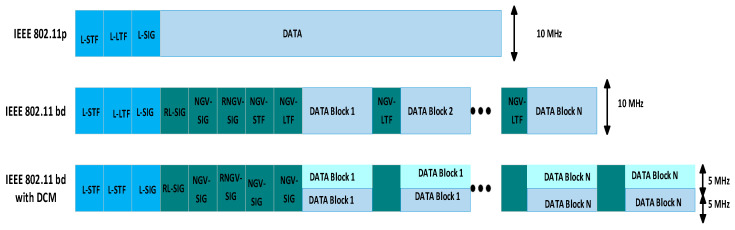
Frame structure of IEEE 802.11p and IEEE 802.11bd.

In addition to utilising the 5.9 GHz band, IEEE 802.11bd also explores the 60 GHz band to enable higher bandwidth. Figure 13 shows the frame structure of IEEE 802.11p and IEEE 802.11bd. It includes the legacy short/long training field (L-STF/LTF). Next-generation vehicles (NGV), the signalling field (L-SIG), and the payload data. The L-STF/LTF facilitates signal detection, frequency offset estimation, and timing synchronisation, while the L-SIG carries header information regarding data length and the modulation and coding scheme (MCS) [5].

## 3. Related Work

Advanced IoV technologies, along with the development of safe, intelligent, and efficient ITS infrastructures, are prominent areas of ongoing research. At the core of ITS are VANETs, which enable communication between vehicles and roadside infrastructure using various technologies, as discussed in the Section 2.

This review specifically focuses on the DSRC, V2V channel model based on the IEEE 802.11p protocol, as shown in Table 3, which is widely used in VANETs due to its low-latency and short-range communication capabilities. IEEE 802.11p-based V2V communication plays a critical role in enhancing passenger safety, preventing accidents, and improving traffic efficiency by enabling direct, real-time information exchange between vehicles without relying on external infrastructure.

Compared with V2I and V2X communication models, V2V offers faster response times, lower latency, lower power consumption, and more efficient data exchange tailored to immediate driving contexts. Its adaptability and scalability make it well-suited to dynamic vehicular environments. Therefore, this review is restricted to VANETs employing DSRC-based IEEE 802.11p V2V communication and aims to explore in depth various channel estimation techniques, highlighting their performance, challenges, and potential improvements for reliable and efficient vehicular communication. By thoroughly examining these techniques, the review seeks to address key limitations of IEEE 802.11p, such as poor performance under high mobility, sensitivity to Doppler effects, limited support for non-line-of-sight scenarios, and a sparse pilot structure that restricts accurate channel estimation by identifying advanced estimation methods that enhance channel reliability, robustness, and adaptability in dynamic vehicular environments.

The Preferred Reporting Items for Systematic Reviews and Meta-Analyses (PRISMA) technique has been used as the systematic review methodology by Moher [55]. IEEE Xplore, Google Scholar, Springer, Elsevier, and Wiley are among the databases searched.

**Figure 14:**
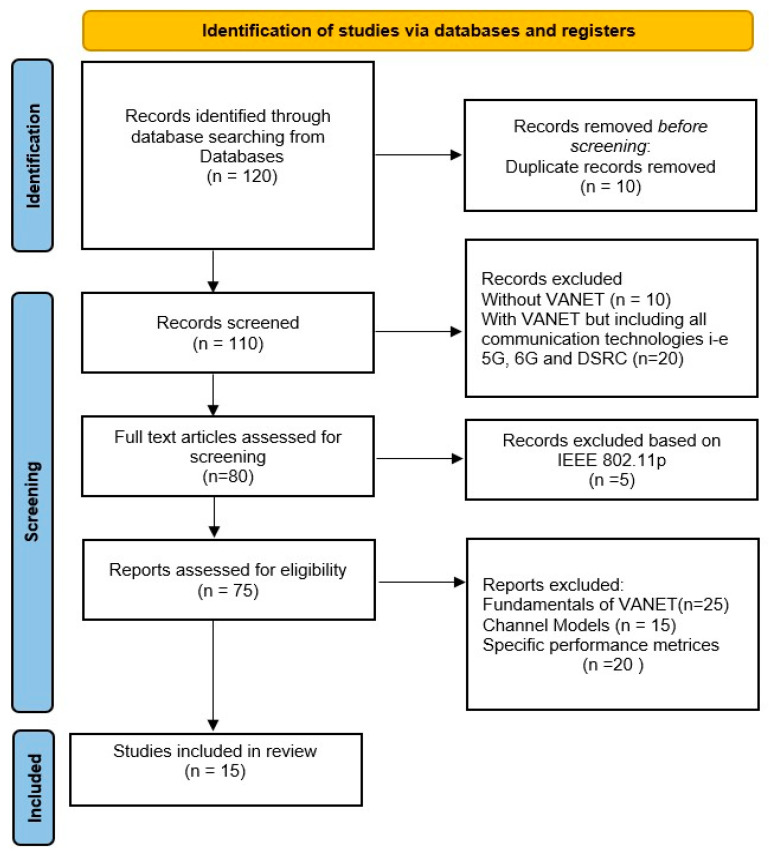
PRISMA Flow diagram. n = No. of articles.

The increasing complexity and variability of wireless communication channels in vehicular networks have necessitated advanced channel estimation techniques. While effective in specific scenarios, conventional methods proposed by [11,33] often struggle in dynamic, high-mobility vehicular networks. As a result, deep learning-based approaches have emerged as a promising solution, offering enhanced performance. In the literature review, the following keywords are used: “Channel Estimation”, “Channel Estimation Technique”, “Deep Learning “, “Dynamic environments”, “High Mobility Scenarios”, “Vehicular Communication”, “Vehicular Technology”, “VANET”, “DSRC”, “IEEE 802.11p” and “IEEE 802.11bd”. As shown in Figure 14, initially, 120 publications are identified from the databases. Ten duplicates are discarded after identification. Furthermore, 10 articles are excluded after skimming for applications unrelated to VANET, and 20 articles are discarded because all wireless technologies, i.e., 5G and 6G, have been explored; however, our research focuses on DSRC. Thus, 80 full-text publications were retrieved and evaluated for eligibility using the exclusion criteria. Moreover, five publications are excluded because they are not related to IEEE 802.11p. Upon further scrutiny, three primary criteria were employed to exclude certain publications from the review: (1) 25 articles are excluded because studies focused solely on the fundamentals of VANET without addressing channel estimation techniques; (2) research utilising generic or non-specific channel models that do not accurately represent V2V communication scenarios due to that reason 15 articles have been omitted and (3) 20 articles are discarded based on publications lacking detailed or relevant performance metrics specific to V2V environments. Following this screening process, 15 high-quality publications were selected for the final review.

### 3.1. Existing Deep Learning Techniques for IEEE 802.11p

**Figure 15:**
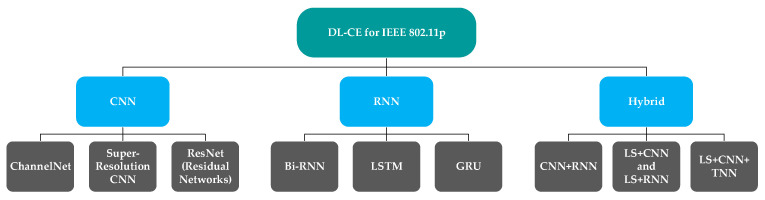
Hierarchical Diagram of DL-Based C.E for IEEE 802.11p.

In the literature, enormous channel estimation techniques have been proposed using the IEEE 802.11p standard. Figure 15 presents a tree diagram of the primary deep learning-based channel estimation techniques comprehensively reviewed for IEEE 802.11p, categorised into CNN-based, RNN-based, and hybrid architectures. The green colour shows an overview of deep learning based channel estimation, and the blues show basic types of deep learning. Last but not least, the grey colour shows different types of each section in detail. The Least Squares (LS) channel estimation technique is one of the simplest and most widely used methods in the IEEE 802.11p standard. LS exploits known pilot symbols to estimate the channel response by minimising the squared error between the received and estimated signals [56,57]. Linear Minimum Mean Square Error (LMMSE) provides improved accuracy as compared to the LS approach in IEEE 802.11p. In addition, it reduces channel estimation errors at low SNR [58]. On the one hand, it relies on many assumptions that limit its performance in high-mobility conditions. Data-Pilot Aided (DPA) utilises the demapped data subcarriers of the previously received OFDM symbol to estimate the channel for the current OFDM symbol [59]. Because of the damping error, it is regarded as unreliable. The Spectral Temporal Averaging (STA) approach, which exploits temporal and frequency correlations across successive received OFDM symbols to update channel estimates for the current symbol, achieves better performance than other conventional schemes in low-SNR regions, but exhibits a significant error floor at high SNRs. However, obtaining such information in real-world scenarios is quite challenging. To address this issue, Kim et al. [43] proposed the Time-Domain Reliable Test Frequency-Domain Interpolation. The Reliability test plays a key role in the TRFI technique. It is performed by equalising the received OFDM symbol using the previously estimated and current channel estimates. Identical equalisation results indicate reliable channel estimates, which are then used to interpolate values for unreliable subcarriers whose equalisation results differ. Awad et al. have presented several tracking algorithms and channel estimation techniques, including decision-directed with time-domain truncation (DD-TT), finite-alphabet with time-domain truncation (FA-TT), and alphabet search with time-domain truncation (AS-TT). Based on the DD channel estimation scheme, a hard-decision algorithm employing a Viterbi decoder has been implemented to achieve low complexity. Traditional channel estimation methods, such as LS and LMMSE, perform well but struggle in dynamic vehicular environments due to limited pilot density and high computational complexity. Techniques such as DPA are prone to error propagation, thereby reducing reliability. Deep learning offers a data-driven alternative that overcomes these limitations by learning channel behaviour directly. It provides robust, accurate, and low-complexity estimation suitable for real-time applications [21,60]. In the following subsections, the literature review explores various deep learning techniques for channel estimation to address the limitations of IEEE 802.11p in vehicular networks, categorising them by model type and application context.

#### 3.1.1. LS-Based Deep Learning Estimation Technique

Both Yang, Y. et al. [61] and Joo, J. et al. [53] have proposed the LS channel estimation technique, which is widely used due to its low computational requirements, making it more efficient than techniques that require no prior information. LS-based DNN channel estimation combines the LS with deep neural networks to improve accuracy. Nevertheless, it addresses the high estimation errors typical of LS methods by learning complex channel characteristics [62].

#### 3.1.2. TFRI-Based Deep Learning Estimation Technique

Time-Frequency Reliable Interpolation (TFRI) is a channel estimation technique designed to address the challenges posed by time-varying, frequency-selective fading, particularly in vehicular communication systems. TFRI identifies reliable pilot and data subcarrier positions in both the time and frequency domains and performs interpolation based on their spatial correlation to reconstruct the channel state information (CSI). When integrated with deep neural networks (DNNs), the TFRI+DNN framework leverages learned representations to better capture underlying time-frequency correlations, significantly improving estimation accuracy compared with conventional interpolation-based methods. However, the use of fixed interpolation patterns limits its adaptability, resulting in performance degradation under high-mobility conditions where channel dynamics change rapidly [51].

#### 3.1.3. STA-Based Deep Learning Estimation Technique

STA is a channel estimation technique that exploits spectral and temporal correlations in wireless channels to improve estimation accuracy while reducing computational complexity. In the frequency domain, STA performs spectral averaging by smoothing channel estimates across adjacent subcarriers, which are likely to experience similar fading conditions due to frequency correlation. Concurrently, temporal averaging is applied across consecutive OFDM symbols to leverage the channel’s time-domain correlation, especially in low-to-moderate mobility scenarios. Unlike conventional fixed-window averaging, STA employs a deep neural network (DNN) to learn adaptive weights for combining spectral and temporal features, thereby enabling the estimator to adapt dynamically to varying channel conditions. This hybrid approach allows a balance between performance and computational efficiency. However, STA’s ability to generalise to highly dynamic environments such as advanced wireless networks characterised by high Doppler spreads, significant frequency selectivity, and complex propagation conditions is limited. In such scenarios, STA may fail to capture long-term dependencies or intricate channel variations, which often necessitate more powerful temporal modelling techniques such as LSTM networks or Transformer-based architectures [52,63]. Temporal averaging using an LSTM-based estimator is investigated to improve performance. However, spectral characteristics may not be captured. The STA technique, when coupled with a DNN, reduces computational complexity; however, it does not apply to advanced wireless networks.

#### 3.1.4. DPA-Based Deep Learning Estimation Technique

Data Pilot-Aided (DPA) channel estimation enhances performance in IEEE 802.11p systems by leveraging both pilot and data subcarriers to improve channel estimation with limited pilot resources. It treats decoded data symbols reliably as auxiliary pilots to refine the channel estimate. When combined with deep learning, DPA models capture complex channel variations through learned temporal and spectral features. However, reducing model complexity often compromises estimation accuracy, highlighting a trade-off between efficiency and performance [60,64].

#### 3.1.5. CNN-Based Deep Learning Estimation Technique

Convolutional Neural Networks (CNNs) have been employed for channel estimation in IEEE 802.11p systems to effectively capture local time-frequency dependencies and enhance estimation accuracy [65,66]. CNNs operate by applying convolutional filters to the input signal, enabling the model to learn spatial features, such as correlations among neighbouring subcarriers and time-domain samples. This capability allows for the network to model the underlying structure of the wireless channel more accurately than traditional methods. However, while CNN-based estimators offer improved performance, their layered architecture and extensive parameter sets increase computational complexity, posing challenges for real-time implementation in vehicular communication environments. In [39,67], Convolutional neural network (CNN)-based channel estimation improves performance, but it also increases computational complexity. In addition, a data pilot-aided temporal Convolutional Networks (TCNs) channel estimation technique is presented for V2V [43]. As a post-processing step, the DPA method can effectively leverage more accurate initial channel estimates from a TCN to estimate the current channel state.

#### 3.1.6. RNN-Based Deep Learning Estimation Technique

Recurrent Neural Networks (RNNs), particularly those employing Gated Recurrent Units (GRUs), have been proposed for channel estimation in vehicular communication systems to capture temporal dependencies in sequential data [50,51]. RNNs process input sequences by maintaining a hidden state that evolves over time, enabling the network to model the temporal dynamics of time-varying wireless channels. GRUs enhance this capability by incorporating gating mechanisms that regulate information flow, thereby mitigating vanishing gradients and improving training efficiency. While RNN-based estimators demonstrate improved performance in moderate mobility scenarios, they are inherently sensitive to noise and exhibit reduced robustness in highly dynamic environments, limiting their applicability under severe channel variations [49,68]. The gated recurrent unit (GRU) optimised by a genetic algorithm (GA) scheme extracts frequency- and time-domain features more accurately and, to suppress noise propagation, incorporates adaptive time processing [46].

#### 3.1.7. Explainable AI-Based Channel Estimation Technique (XAI-CHEST)

X-AI is critical for autonomous driving, 6G and vehicular communications because it ensures interpretability and trustworthiness. X-AI refers to black-box models that help to identify relevant inputs in decision-making [69]. XAI-CHEST is a novel perturbation-based feature selection framework that outperforms conventional feature selection methods, such as local interpretable model-agnostic explanations (LIME) and Shapley additive explanations (SHAP), in terms of interpretability and complexity [42]. In addition, XAI-CHEST is an optimised model that achieves lower BER than LIME and SHAP by selecting the most relevant subcarriers and avoiding irrelevant ones.

## 4. Discussion

The challenges, issues, and limitations correlated with traditional and Deep learning-based channel estimation techniques for the IEEE 802.11p protocol have been explored.

Traditional estimation methods, such as LS, MMSE, STA, and TFRI, are simple and less computationally complex but fail to account for rapid channel variations in highly dynamic environments, e.g., V2V scenarios. LS is highly noise-sensitive and inaccurate with the sparse pilot structure of IEEE 802.11p. MMSE depends on prior channel statistics, which quickly become outdated when vehicles move at high speeds. STA and TFRI depend on temporal or frequency correlations, but these assumptions break down under large Doppler shifts, where the channel coherence time is shorter than the pilot spacing. As a result, these estimators rely on stale or insufficient information, resulting in low accuracy and degraded performance in rapidly varying vehicular channels. For example, Deep learning-based channel estimation methods enhance channel performance by learning complex temporal and spectral correlations. Techniques such as STA-DNN, TRFI-DNN, Bi-RNN, SR-ConvLSTM, and TCN+DPA achieve significant improvements in BER and MSE, especially at higher SNR. However, these enhancements increase computational complexity due to multi-layered neural architectures, high-dimensional parameterisation, and iterative training methods, which are key constraints in V2V communication, where latency is the primary concern. This article examines 15 research articles on deep learning-based channel estimation techniques, as listed in Table 3, with a focus on IEEE 802.11p and V2V channel models. Moreover, other hybrid techniques have been analysed.

The channel is estimated using a hybrid LSTM model based on IEEE 802.11p [70,71]. LSTM-based methods suffer from significant computational. Hybrid orthogonal time-frequency space (OTFS) modulation-based techniques and Hybrid RNN (Bi RNN) approaches [47,48] are presented to reduce computational complexity and improve robustness. However, RNN-based techniques alone may not be sufficient to capture both spectral and temporal features. Thus, hybrid CNN, RNN, and Bi-RNN techniques are implemented; however, this approach is susceptible to noise. Zhang et al. [72] predicted that the channel will use a hybrid Transformer-based technique for vehicular Communication. Again, there is a need to reduce latency and improve accuracy. Analysing existing deep learning-based channel estimation schemes for IEEE 802.11p makes balancing trade-offs among all performance factors challenging. Thus, the objective of future research work should be to prioritise the development of a channel estimation frameworks that integrate lightweight DL models with traditional techniques, optimised for edge deployment. Additionally, context-aware algorithms that dynamically adjust model complexity based on vehicular mobility and channel conditions will be essential to achieving a practical balance between accuracy, complexity, and latency in next-generation V2X systems. Moreover, to propose an IEEE 802.11bd protocol that is more advanced than IEEE 802.1p discussed in the above section. The IEEE 802.11bd standard introduces key enhancements, such as midambles for tracking and dual-carrier modulation for frequency diversity, that collectively support more robust, adaptive channel estimation. The IEEE 802.11bd standard provides a more suitable framework for intelligent channel estimation, with structural and coding enhancements that enable adaptive, low-latency solutions. By leveraging these advancements, future V2V systems can better balance trade-offs among estimation performance, real-time responsiveness, and system efficiency, advancing the broader goal of safe, reliable, and intelligent transportation networks.

## 5. Conclusions and Future Direction Perspectives

The potential of deep learning-based methods to address the limitations of IEEE 802.11p has been reviewed comprehensively. DL-based channel estimation techniques offer substantial improvements in estimation accuracy and reduced computational complexity. However, balancing the trade-off among these performance metrics, accuracy, computational complexity, and delay, is challenging for existing methods in IEEE 802.11p. In V2V communications, latency is a key factor that needs to be reduced. Therefore, there is a need to develop a technique that addresses these major issues, and IEEE 802.11bd should be considered for future work to support a reliable, intelligent transportation system. The TNN techniques have been aligned with the features of the IEEE 802.11bd protocols. TNNs enhance robustness in high-mobility environments by modelling fast-changing channels. At the same time, their attention mechanisms exploit midamble positions for more accurate symbol recovery, leading to improved BER and NMSE under dynamic environments. In addition, TNNs enable end-to-end learning by jointly optimising synchronisation, equalisation, and decoding, thereby simplifying receiver design and improving overall performance. Lightweight variations can further capture spectral correlations efficiently while meeting the low-latency requirements of V2V systems. Overall, IEEE 802.11bd provides a more suitable framework for deep learning–based channel estimation and leveraging these enhancements can help future V2V systems achieve an effective trade-off between channel performance and system efficiency, contributing to safe, reliable, and intelligent transportation.

## Figures and Tables

**Table 1:** IEEE 802.11p Physical layer specification [39].

Parameters	Values
Bandwidth	10 MHz
FFT size	64
FFT period	6.4 µsec
Guard interval duration	1.6 µsec
Symbol duration	8 µsec
Total subcarriers	64
Pilot subcarriers	4
Data subcarriers	48
Null subcarriers	12
Subcarrier spacing	156.25 kHz
Modulation scheme	QAM
Data rate	3–27 Mbps

**Table 2:** Feature comparison of IEEE 802.11p and IEEE 802.11bd [35].

Features	IEEE 802.11p	IEEE 802.11bd
Channel Bandwidth	10 MHz	10 MHz (20 MHz)
Subcarrier spacing	156.25 kHz	156.25 kHz
Channel coding	Convolutional	LDPC
Lowest MCS	BPSK (1/2)	BPSK (1/2)
Highest MCS	64-QAM (5/6)	256-QAM
Preamble and Header	5 symbols/40 µs	10 symbols/80 µs
Doppler Recovery	Preamble only	High-density mid bands
Range extension	Not supported	Optional
Supported relative speed	252 km/h	500 km/h

**Table 3:** Systematic Review of Deep Learning Based Channel Estimation Schemes.

Author	Year	Techniques	Why It Matters	Recent Extensions
Gizzini et al.	2025 [42]	XAI-CHEST	Improves accuracy and interpretability while reducing complexity, a crucial factor for explainable and trustworthy V2X systems.	Explainable attention maps enhance reliability in safety-critical scenarios; the XAI trend grows for real-time vehicular use.
Ngorima et al.	2025 [43]	TCN+DPA	Achieves strong BER performance, but CNN-based estimators remain computationally heavy for embedded vehicular devices.	TCN, with dual-path attention, delivers low-latency BER gains while balancing performance with resource constraints.
Shukla Devesh et al.	2025 [44]	DNN	Demonstrates good BER across multiple scenarios, but switching between offline and online training degrades robustness.	Federated and continual learning approaches improve adaptability across urban, rural, and highway environments.
Luan et al.	2023 [45]	Encoder–decoder Architecture	Captures time–frequency dynamics better than CNNs but still suffers from latency in high mobility.	Transformer encoder–decoder (e.g., AdaFortiTran, OTFS-Transformer) shows superior Doppler tracking with midamble-aided estimation.
Xiaojun	2025 [46]	GA-GRU-AT	Performs well under higher-order modulations but depends on offline GA training, limiting adaptability.	Adaptive GA and RL-based hyperparameter tuning allow real-time operation without retraining.
Gizzini et al.	2023 [47]	RNN (Bi-GRU)	Handles sequential fading, but suffers from doubly selective channels (Doppler + multipath).	Lightweight and sparse GRUs cut FLOPs while maintaining accuracy; hybrid GRU-attention improves URLLC V2X reliability.
Gizzini et al.	2023 [48]	RNN (GRU)	Reliable but computationally demanding; complexity still hinders deployment.	Efficient GRU + attention integration provides greater robustness to fast fading.
Hou et al.	2022 [49]	GRU	Achieves BER gains but is limited by reliance on Tapped Delay Line (TDL) channel models.	Pilot-aided GRU learning boosts robustness; however, CNN-Transformer hybrids outperform GRUs in dynamic V2V.
Gizzini et al.	2021 [39]	CNN Aided Weighted Interpolation	Strong at capturing local channel features, but CNNs demand a high computational cost.	Shift toward hybrid models, e.g., ConvTrans-ResNet, that efficiently capture both local and global features.
Gizzini et al.	2021 [50]	TA+LSTM	Improves learning in high-mobility scenarios by combining temporal attention and memory to capture time-varying channel characteristics better.	Transformer-based temporal attention models have replaced LSTM models, improved parallelisation, and reduced latency.
Gizzini et al.	2020 [51]	TFRI+DNN	Utilizes time–frequency relationships to improve pilot efficiency and learning nonlinear channel variations.	Hybrid models optimise feature extraction across the time and frequency domains for more precise estimation.
Gizzini et al.	2020 [38]	STA+DNN	Focuses on spatial–temporal averaging to achieve more stable estimation in the presence of multipath fading.	Adaptive spatial–temporal attention networks dynamically adjust coefficients using data-driven optimization for non-stationary channels.
Han et al.	2019 [52]	DPA+Autoencoder	Uses deep power allocation and AE-based reconstruction to enhance BER and robustness in nonlinear channels.	Lightweight autoencoder designs and variational AE architectures now reduce complexity and demapping errors.
Joo et al.	2019 [53]	LS+STM	To improve the temporal prediction of fading effects, the LS is integrated with an LSTM.	Reliability and applicability.
Awad et al.	2018 [54]	DD+Viterbi decoder	To achieve low-complexity symbol detection, decision-directed estimation with classical decoding has been employed.	DNN-assisted Viterbi decoders for adaptive modulation and higher-order QAM with lower computational cost have been integrated into recent advancements.

## Data Availability

Not applicable.
